# Rationale and protocol of the LEAD 2.0 study: a randomized controlled trial assessing the feasibility of a virtually delivered 6-month exercise and nutrition intervention in older adults with subjective cognitive decline (SCD)

**DOI:** 10.1186/s40814-025-01626-4

**Published:** 2025-05-10

**Authors:** Bobby Neudorf, Noah Koblinsky, Krista Power, Malcom Binns, Alexandra J. Fiocco, Shlomit Rotenberg, Susan Marzolini, Paul Oh, Jane Thornton, Fatim Ajwani, Kylie Sullivan, Stéphanie Chevalier, Caryl Russell, Guylaine Ferland, Nicole D. Anderson, Laura E. Middleton

**Affiliations:** 1 Department of Kinesiology, University of Waterloo, Waterloo, ON Canada; 2 Rotman Research Institute, Baycrest Academy for Research and Education, Toronto, ON Canada; 3 School of Nutrition Sciences, University of Ottawa, Ottawa, ON Canada; 4 Department of Psychology, Toronto Metropolitan University, Toronto, ON Canada; 5 Department of Occupational Science and Occupational Therapy, University of Toronto, Toronto, ON Canada; 6 Kite Research Institute, University Health Network, Toronto Rehabilitation Institute, Toronto, ON Canada; 7 Rehabilitation Sciences Institute, University of Toronto, Toronto, ON Canada; 8 Department of Family Medicine, Schulich School of Medicine and Dentistry, University of Western Ontario, London, ON Canada; 9 School of Human Nutrition, Mcgill University and Research Instituteof the, Mcgill University Health Centreaq , Montreal, QC Canada; 10 Département de Nutrition, Montreal Heart Institute Research Centreand, Université de Montréal, Montréal, QC Canada; 11 Departments of Psychology & Psychiatry, University of Toronto, Toronto, ON Canada

**Keywords:** Cognition, Exercise, Diet, Feasibility, RCT, Virtual

## Abstract

**Background:**

With growing prevalence of dementia worldwide, dementia risk reduction is a key interest of the World Health Organization’s Global Dementia Action Plan. Subjective cognitive decline (SCD) is a prominent predictor of future dementia diagnosis. Therefore, people with SCD are an important group for dementia prevention intervention. Exercise and healthy diet are associated with a 30–60% decrease in dementia risk in longitudinal studies. Technological advances yield the potential of trials that deliver lifestyle interventions virtually, reaching people in a wide geographical spread. However, the feasibility of large-scale virtual trials still needs to be established.

**Objective:**

This trial aims to examine the feasibility of a factorial randomized controlled trial exploring a 6-month virtual, exercise and healthy diet intervention. Secondary objectives will examine whether feasibility outcomes vary by gender or technology access.

**Methods:**

We will recruit 140 older adults (65 + years) with SCD, who will receive a combination of Aerobic and Resistance Exercise (EX) or Stretching and Toning (STRETCH) and either Diet Counseling (DIET) or Brain Health Education (ED). Participants will be randomized to four weekly hours of one of four intervention arms: (1) EX and DIET; (2) EX and ED; (3) STRETCH and DIET; or (4) STRETCH and ED. EX will include moderate intensity aerobic and resistance training. DIET will instruct participants in brain healthy food choices. Assessments will be performed virtually at baseline, 6 months (post-intervention), and 12 months. Feasibility will be measured by recruitment rate, adherence, and retention.

**Discussion:**

Established feasibility will set the stage for a definitive trial. Feasibility results will also inform future virtual programs/services. In the long-term, if the interventions are feasible and beneficial, this intervention model could scale up and spread quickly to reach at-risk individuals for the purpose of dementia risk reduction.

**Trial registration:**

The Lifestyle, Exercise, and Diet (LEAD 2.0) study is registered with the US National Institutes of Health clinical trials registry (ClinicalTrials.gov identifier NCT06078748). This report complies with the Standard Protocol Items: Recommendations for Interventional Trials (SPIRIT) statement.

**Supplementary Information:**

The online version contains supplementary material available at 10.1186/s40814-025-01626-4.

## Background

More than 55 million individuals worldwide are living with dementia [1]. In 2019, the global cost of dementia reached 1.3 trillion US dollars, which is expected to rise to 2 trillion by 2030 [1, 2]. Dementia risk reduction is a key action area of the World Health Organization’s Global action plan on the public health response to dementia 2017–2025 [2]. It is estimated that 40% of dementia cases could be prevented by addressing modifiable risk factors [3].

Subjective cognitive decline (SCD), where people perceive cognitive changes but continue to score within age and education norms on cognitive tests [4–7], is the most prominent feature predicting future cognitive impairment [8]. A systematic review concluded that those with SCD have double the risk of dementia compared to those without cognitive concerns, and 6.5% of people with SCD convert to mild cognitive impairment (MCI) or dementia each year [9]. Individuals with SCD are generally not managed within the healthcare system due to a lack of noticeable cognitive deficits [10]. As such, people with SCD may be an opportune group to target with interventions to improve lifestyle, cognition, and function before irreversible cognitive changes occur [4–9], as 95% of individuals with SCD report difficulty incorporating exercise and healthy eating into their lifestyles [11].

Exercise and healthy eating are associated with lower dementia risk (30–60%) in longitudinal studies [12–15] and appear to have the most consistent influence on executive function [16–18]. While the synergistic effects of exercise and healthy eating on cognition have yet to be explored together, they have been studied separately extensively. A systematic review of 39 studies conducted from 1989 to 2016 suggests moderate-intensity exercise has significant interaction effects on executive function, memory, and working memory among adults over the age of 50 [16], with several studies demonstrating that cognitive changes are underpinned by changes in brain structure and function [19–22]. While several systematic reviews of RCTs examining dietary interventions conducted from 2000 to 2016 have shown to be inconsistent with highly variable effect sizes [17, 18], other reviews have demonstrated that higher adherence to the Mediterranean, DASH, and MIND diets are associated with less cognitive decline and a decreased risk of developing mild cognitive impairment and dementia [23–25]. A possible explanation for inconsistencies among diet interventions is that they often rely on infrequent (bi-weekly to quarterly) education [17, 18, 26–30], which may be insufficient to promote predictable and sustained eating changes [31]. Interventions that integrate skill-training to support behavioral change could increase the efficacy of diet interventions in the short-term, and exercise and diet changes in the long-term (following the intervention), when it is common for exercise levels to drop below post-intervention levels or to baseline levels [32].

A new opportunity is the use of virtual technologies to expand the reach of such trials. While access to high-speed internet among rural populations is substantially lower than urban populations (45.6% vs. 98.6%), government initiatives to expand rural broadband connection are on track to reach 95% of the Canadian population by 2026 [33, 34]. The COVID-19 pandemic has also positively contributed to the virtual delivery of lifestyle interventions, expanding the geographical reach of interventions in research and in practice, while technology use among older adults has accelerated as 75% of older adults now feel confident using current technology [35]. Furthermore, virtual or online programs limit the barriers to participation in in-person programs, for example, the need for transportation and navigating cities and facilities that are not always accessible [36–38]. Overall, this suggests a sizable audience of older adults who could and may be inclined to access virtual group interventions.

In the long-term, the research team’s aim is to conduct a fully powered, virtual, factorial RCT, which will require 404 participants over 3.5 years. The planned future primary outcome will be executive function. Although, before embarking on such a large, expensive trial, it is important to first determine that such a trial will be feasible. While several studies have demonstrated the feasibility of virtual exercise and education interventions [39–42], including those for individuals with cognitive impairment [36, 43, 44], there is no existing literature to support the feasibility of a 6-month, virtual RCT with follow-up at 12 months and compliance to an active control group that is time/frequency matched to intervention groups.

Overall, the ultimate aim of this study is to determine the feasibility of an entirely virtual factorial RCT; a 6-month virtual exercise and nutrition trial among older adults with subjective cognitive decline. This factorial RCT will examine the independent and combined effects of aerobic and resistance exercise versus stretching control, and healthy diet counseling versus brain health education control on executive function among people with SCD.

### Objectives and hypothesis

The primary objective of this study is to determine the feasibility of a 6-month virtual exercise and nutrition trial among older adults with SCD. Feasibility will be based on the following pre-determined criteria: (1) Recruitment: ≥ 9 participants/month over 1.25 years; (2) Adherence: (a) ≥ 75% of Aerobic and Resistance Exercise (EX) sessions and ≥ 75% of Diet Counseling (DIET) sessions are completed; (b) improvement in diet quality at 6 months compared to baseline; (3) Retention: ≥ 80% of randomized participants complete the 6-month assessment of executive function (future primary outcome).

Secondary objectives of this study are to (1) examine whether feasibility outcomes vary by gender (as exercise and diet preferences may vary by gender [45, 46] or technology access (whether or not the participant needs a study-provided tablet or internet access for the intervention); (2) inform the future large-scale trial, including (i) the future primary outcome: an executive function composite derived from Cambridge Brain Sciences on-line assessments [47] and (ii) the secondary outcomes: memory (where delayed recall is also among the domains most affected by exercise and diet) [16–18], physical function, quality of life, diet quality, physical activity, and waist circumference, (iii) maintenance of changes in executive function, diet quality, and physical activity at 12 months (6 months post-intervention); (3) examine differences in observed effect sizes by sex, where exercise may have greater benefits to cognitive function among females relative to males [48]; and (4) examine whether EX and DIET are more effective in individuals with executive SCD versus non-executive SCD.

## Methods

### Trial design

**Fig. 1:**
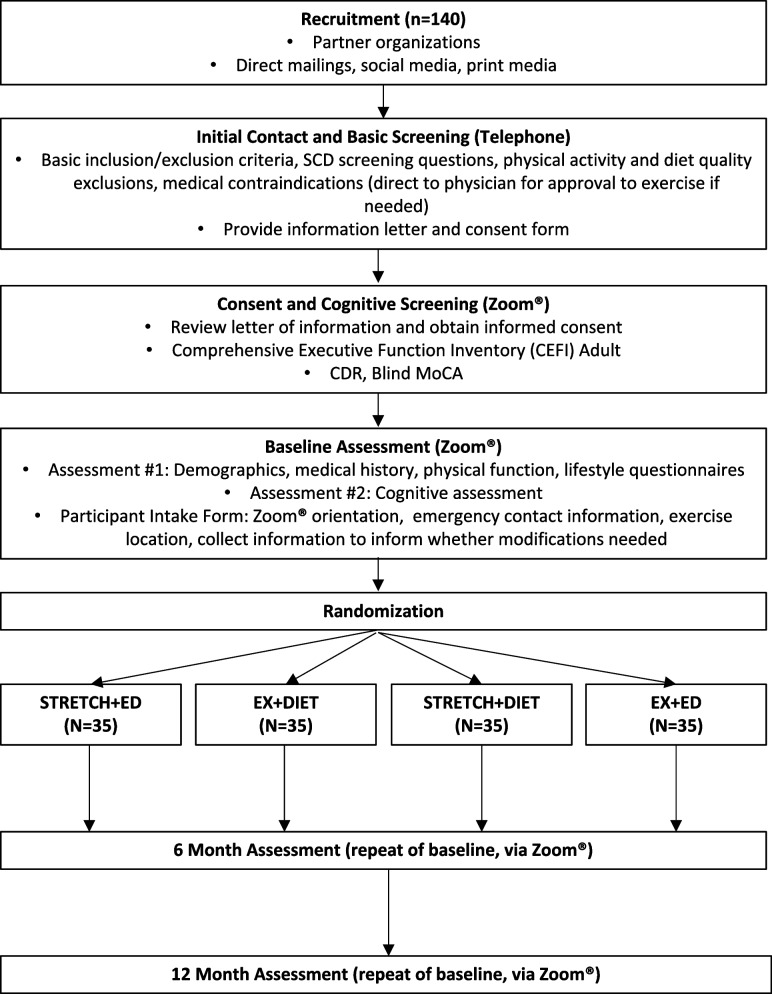
Study flow chart

We will recruit 140 older adults with SCD to this feasibility, factorial RCT of exercise and diet interventions. Following initial contact and consent, participants will attend a virtual screening visit to further assess eligibility. If deemed eligible, participants will attend two baseline virtual assessments which will index medical history and demographic information, and assess thinking abilities, physical function, and quality of life. After participants complete both baseline assessments, they will be randomized into one of four intervention arms and will receive a combination of two of the following: Aerobic and Resistance Exercise (EX); Diet Counseling (DIET); Brain Health Education (ED); and/or Stretching and Toning (STRETCH). The four intervention arms include (1) EX and DIET; (2) EX and ED; (3) STRETCH and DIET; or (4) STRETCH and ED. The two virtual assessments completed at baseline will be repeated at post-intervention (6 months) and follow-up (12 months). See Fig. 1 for the study flow chart.

This study has received ethics approval by the University of Waterloo Research Ethics Board (#44,616) and by the Baycrest Research Ethics Board (#23–24). The Lifestyle, Exercise, and Diet (LEAD 2.0) study is registered with the US National Institutes of Health clinical trials registry (ClinicalTrials.gov identifier NCT06078748). This report complies with the Standard Protocol Items: Recommendations for Interventional Trials (SPIRIT) statement.

### Study setting

This study will be conducted virtually, allowing for the recruitment of participants across four provinces (Saskatchewan, Manitoba, Ontario, and Quebec) that are within a 2-h time zone. Intervention and assessment sessions will be conducted over the Zoom® Healthcare platform. The study sites coordinating and conducting assessment and intervention sessions are the University of Waterloo, Waterloo, Ontario, Canada and Baycrest Academy for Research and Education, Toronto, Ontario, Canada. Participants will join the sessions using an electronic device (computer, tablet) from a place of their choosing, but most often from their home.

### Eligibility criteria

Participants will be 65–85 years old and meet criteria for SCD by (a) answering Yes to both of the following questions (i) Do you feel like your memory or thinking is becoming worse? and (ii) Does this worry you? [7, 49] and (b) having no objective cognitive impairment as indicated by (i) a global Clinical Dementia Rating (CDR) of 0.5 or lower and (ii) a Blind Montreal Cognitive Assessment (MoCA) total score of > 17. Participants will also meet the following criteria:

1. Be able to communicate in English
2. Be residents of Saskatchewan, Manitoba, Ontario, or Quebec
3. Have low physical activity levels (< 75 min/week of aerobic moderate to vigorous physical activity on the Canadian Society for Exercise Physiology’s (CSEP) Get Active Questionnaire [50])
4. Be screened safe to participate in moderate exercise using the CSEP Get Active Questionnaire [50] or physician approval to engage in moderate intensity exercise without in-person supervision
5. Have low diet quality (below Canadian older adults’ median intake of fruits, vegetables, nuts, and fish, reported using our Diet Screening Questionnaire targeting the Brain Health Food Guide criteria, used in the LEAD 1.0 pilot [51])
6. Be able to participate remotely (i.e., availability of, or ability/willingness to adopt a computer or tablet with high-speed internet/data networks)

Participants will be excluded if they have a history of dementia, stroke, or other significant known chronic brain disease, have had chemotherapy or radiation to the head/neck in the past year, have sensory impairments that would impede participation in the intervention or assessments, have current major or unmanaged psychiatric disorder or history of hospitalization for a psychiatric disorder, have ongoing alcohol or drug abuse that in the opinion of the investigator may interfere with the participant’s ability to comply with the study procedures, or have contraindications for exercise as determined by the CSEP Get Active Questionnaire [50].

To overcome barriers to technology access, tablets with high-speed data packages will be provided to older adults who do not own a computer or tablet and do not have internet access. Participants will also be provided with equipment needed for videoconferencing (i.e., webcam, microphone) if they do not already own it and will be provided with an orientation to Zoom® if they are not familiar with it. Despite this, we recognize that barriers may still remain, such as in rural areas that do not have adequate data coverage for videoconferencing. Although, it is estimated that 76% of Canadians have sufficient data speeds to support videoconferencing, with the Government of Canada announcing plans to make connectivity sufficient for web-conferencing available to 95% of Canadians by 2026 [33].

Participants will also complete the Comprehensive Executive Function Inventory for Adults (CEFI-Adult) during the screening process. The research team originally intended to screen participants for executive SCD but decided to screen for general SCD 1 month into the study due to a low recruitment rate. In secondary analyses, we will determine whether outcomes differ between those with or without executive SCD. The presence of executive SCD is indicative of CEFI-Adult scores ≥ 1 SD below age, sex, and education-based norms.

### Recruitment process and strategy

Recruitment of participants began in July 2023 and is expected to be completed in December 2024. The target is to recruit 140 participants across 5 waves, using a variety of recruitment strategies to increase the likelihood of reaching diverse older adults: directed mailings, social media, websites, radio/newspaper advertisements, research and community organization email lists, partner websites and centers, and outreach to physician/seniors’ groups. Participants will also be recruited from targeted research pools and partners email lists including the investigators’ research institutes, the Canadian Consortium for Neurodegeneration in Aging (CCNA), and other project partners.

Potential participants will be contacted by telephone by a member of the research team who will go through the recruitment script to describe the study and screen for eligibility. During this call, a brief assessment for SCD will be conducted. Eligible participants will be sent an electronic version of the letter of information and consent form and will schedule their second screening visit to rule out objective cognitive impairment. All eligible participants will then complete virtual assessments 1 and 2 within 6 weeks of their program start date. Once the assessments are completed, participants will be randomized into one of the four study arms. See Table 1 for an outline of the assessments and timeline.

**Table 1:** Schedule of study assessments

Timepoint	Study timeline			
	Enrollment	Baseline	Post-Int	Follow-up
	− t_1_	t_1_	t_2_	6 months after t_2_ assessment, t_3_
Screening and enrollment				
Screening visit #1	X			
Informed content	X			
Screening visit #2	X			
Virtual assessment 1				
Sociodemographic information		X		
Language proficiency and acquisition		X		
Medical and mental health history		X	X	X
Current medications		X	X	X
Technology proficiency survey		X		
PASE		X	X	X
EPSA		X	X	X
Waist circumference		X	X	X
5 × sit to stand test		X	X	X
SF-36		X	X	X
CES-D		X	X	X
GAI		X	X	X
Virtual assessment 2				
CBS Double Trouble Stroop Test		X	X	X
CBS Monkey Ladder		X	X	X
CBS Feature Match		X	X	X
CBS Paired Associates task		X	X	X
RAVLT		X	X	X

*PASE *Physical Activity Scale for the Elderly, *EPSA *Eating Pattern Self-Assessment, *SF-36 *Short-Form 36, *CES-D *Center for Epidemiological Studies Depression Scale, *GAI *General Anxiety Inventory, *CBS *Cambridge Brain Sciences, *RAVLT *Rey Auditory Verbal Learning Test

### Randomization and blinding procedure

Participants will be randomly assigned (1:1:1:1) after baseline assessments, stratified by gender (man/woman, and if sufficient in number, non-binary/self-described) and balanced within blocks of variable size (4 or 8) to avoid predictable participant allocation. Eligible partnered couples will be randomized together. The randomization sequence will be computer-generated and maintained by an assistant not affiliated with the study. Once baseline assessments are complete, the research coordinator will provide the participant ID to the randomization assistant who will inform the coordinator of the group allocation, which will be communicated to the participant. Participants will start the trial in waves of 24 to 32 (6 to 9 people per group; every 3 months) to ensure sufficient sample size for group dynamics and social support.

Assessors will be blinded to group assignment and participants will be asked not to mention their group assignment to assessors. Standardized instructions will be provided for assessments. To keep participants blinded from the study hypotheses and to minimize group effect expectations, the content of the intervention and the wording of recruitment documents and consent forms will not convey the differences between EX and STRETCH. Participants will be told that the groups will differ in the type of brain health education that is provided (DIET versus ED).

### Trial interventions

Participants will engage in two virtual group sessions (2 h and 1 h in length) and one independent session (1 h) per week. The breakdown of each group’s weekly sessions is shown in Table 2. The balance across intervention components varies between the first 4 months and last 2 months of the trial, whereby the Goal-Setting component will primarily focus on DIET in the first 4 months and EX in the last 2 months. Furthermore, participants will be encouraged to integrate more of their exercise within community settings (i.e., join an exercise class rather than relying on the study video) in the last 2 months of the intervention.

**Table 2:** Intervention time by study arm

Phase 1: Months 1–4					
Group	Group session 1 (30 min)		Group session 1 (90 min)	Group session 2 (1 h)	Independent session (video) (1 h)
EX + DIET	EX		DIET + GOAL	EX	EX
EX + ED	EX		ED	EX	EX
STRETCH + DIET	STRETCH		DIET + GOAL	STRETCH	STRETCH
STRETCH + ED	STRETCH		ED	STRETCH	STRETCH
Phase 2: Months 5–6					
Group	Group session 1 (30 min)	Group session 1 (90 min)		Group session 2 (1 h)	Independent session (1 h)
EX + DIET	EX	EX GOAL + DIET		EX	EX (home/community)
EX + ED	EX	EX GOAL + ED		EX	EX (home/community)
STRETCH + DIET	STRETCH	ED + DIET		STRETCH	STRETCH (video)
STRETCH + ED	STRETCH	ED		STRETCH	STRETCH (video)

Orientation: Prior to the first intervention session, participants will meet with the instructor in a one-on-one Zoom® call. Participants will receive a summary of how to use Zoom® (e.g., opening Zoom®, connecting video and audio, settings, mute/unmute, using the chat) and be provided with a telephone number/email to contact a study volunteer for technology help during classes. The instructor will gather the participant’s emergency contact or any other relevant information that may be necessary in case of an emergency or to inform exercise modifications (i.e., medical conditions, symptoms, medications). They will then help the participant choose a safe exercise location and a good position for their device. Participants will be encouraged to use a bright room with space for exercise and a hard non-slippery surface free from clutter and any tripping hazards. The device (computer or tablet) should be placed in a position where the instructor will be able to see them exercising. The participant will be asked to mark off the placement area so they know exactly where to put their device each time. The instructor will then go through a checklist of what to wear (running shoes and comfortable clothing) and what to have with them (phone within reach, water bottle, chair, resistance bands, and exercise mat). Prior to the intervention starting, participants will be mailed resistance bands and an exercise mat.

The instructor will also describe the general class routine (length, format, group-based) and discuss safety information while exercising (including working within own limitations, as well as to immediately notify instructor if feeling lightheaded, dizzy, unwell, chest pain, muscle pain). The emergency procedures will also be discussed, and participants will receive a copy of this procedure via email. Participants will be asked to leave their webcams on and to notify the instructor with a wave if they are leaving the screen for a non-medical reason. If participants leave the screen without notice, the instructor will call them and then call emergency services if an emergency is suspected.

The instructor will also complete the Participant Intake Form (see Supplementary Material 2) with each participant. The instructor will use this information (along with the recommendations made by a participant’s physician, if applicable), to recommend that the participant complete EX/STRETCH seated or standing.

EX intervention: Participants in the exercise intervention (EX + DIET or EX + ED) will complete 2.5 h of moderate intensity aerobic, resistance, and balance exercise per week. EX will be instructed by a Certified Exercise Physiologist (CEP) or a Registered Kinesiologist with a volunteer also being present for assistance.

At the start of each EX session, the instructor will welcome the group and the volunteer will record attendance. The volunteer will remind participants of safety precautions and will confirm that participants have a phone within arm’s reach. The 30-min EX session will include a 5-min warm-up, 20 min of aerobic training, and 5 min of balance training and cool-down. The 60-min EX session will include a 5-min warm-up, 20–25 min of aerobic training, 20–25 min of resistance training, 5 min of balance training, and a 5-min cool-down. The virtual video session will be pre-recorded, have similar composition to the 60-min EX session, and will be led by the same instructor. In months 5 and 6 of the intervention, participants will be asked to replace the video session with home- or community-based exercise of their choosing, with encouragement to match the intensity and duration of the video sessions.

Aerobic exercise will include exercises that are appropriate for older adults and can be completed at home, such as marching on the spot, walking forward/backwards, side stepping, and heel/toe taps. Resistance exercises will follow the Cardiac College recommended resistance exercises for older adults [52], which target major muscle groups including the legs, chest, back, shoulders, and core, and use body weight, home objects, and study-provided resistance bands to increase challenge. Participants can complete exercises seated or standing, according to their own preference or based on recommendation by the instructor (along with the recommendations made by a participant’s physician, if applicable). The difficulty of exercise sessions will be progressed over the study, adapted to the range of fitness, strength, and ability. Participants will be instructed to target an intensity of 3 or less during the first several weeks, and 4 on the 10-point RPE scale during the remainder of the program (equivalent to moderate intensity; rated on the last 2 to 3 repetitions for resistance training) [53].

STRETCH control: The STRETCH sessions will be time/frequency-matched to EX sessions (2.5 h/week: 1.5 h in virtual group sessions; 1 h in virtual video session) to control for social aspects and placebo effects of EX. The STRETCH session will start in the same way as the EX session. Each 30-min STRETCH session will include a 5-min warm-up, 5 min of balance exercises, and 20 min of stretching and toning. Each 60-min STRETCH session will include a 5-min warm-up, 5 min of balance training, and 50 min of stretching and toning. Difficulty will not be progressed. STRETCH sessions will also be led by a CEP or Registered Kinesiologist, with a volunteer present for assistance. Video sessions will be pre-recorded by the same instructor. Again, participants can complete exercises seated or standing, according to their own preference or based on recommendations by the instructor (along with the recommendations made by a participant’s physician, if applicable).

DIET intervention: The DIET intervention includes one session per week, delivered by a Registered Dietitian. The weekly DIET session will be 90 min during months 1 to 4, and 30 min in months 5 and 6. The DIET intervention was developed by the research team, emphasizing foods identified as supporting executive function, memory, and other cognitive abilities in prior research and included in the Brain Health Food Guide [54] (see Supplementary Material 4). DIET integrates recommendations from the Mediterranean diet and the DASH diet as well as individual foods that have been associated with brain health [55–58]. Beneficial food groups include vegetables (and raw/leafy green vegetables, cruciferous vegetables particularly), fruits (berries particularly), unsalted nuts (walnuts particularly) or natural nut butters, fish or seafood, fatty fish, beans, and legumes. Foods to limit include meat and poultry, red or processed meat, butter, cream, or high fat dairy spreads, white bread, and processed foods. Individuals in the DIET group will fill out the Eating Pattern Self-Assessment monthly to help with goal setting.

ED control: The ED control program was adapted from the placebo condition of the Discovery program used by the Canadian Consortium on Neurodegeneration in Aging (CCNA) sub-study ENGAGE (NCT#03271190) for their placebo intervention [59], which also served as a diet placebo condition in the LEAD 1.0 pilot [51]. These classes are designed to be of equal intensity and social interaction/engagement compared to the DIET sessions and have been manualized to ensure consistency between instructors. During these sessions, participants will engage in group discussion and receive information on the brain and cognitive processes, the effect of age on cognition, and tips to promote healthy aging. This will include lectures, watching documentaries, and participating in various games.

Goal-Setting: All arms that receive DIET and/or EX interventions will also receive the Goal-Setting training, with goals focused on diet and/or exercise [60, 61]. This meta-cognitive skill-acquisition intervention is based on the Cognitive Orientation to daily Occupational Performance (CO-OP) approach [60], participants’ goal setting, behavioral self-monitoring, and problem-solving skills, three behavior change techniques commonly and effectively used to promote physical activity and improve dietary behaviors among older adults [62–65]. Participants are taught to use a global strategy (Goal-Plan-Do-Check) to facilitate individualized exercise or diet goal setting and develop plans to achieve them, through an iterative problem-solving process [60, 61]. To support skill development, the goal setting intervention will use guided discovery methods, a series of hierarchical questions or guiding statements that lead the participants to discover and articulate their thoughts or actions [60].

Participants will set two individualized goals that align with exercise and/or diet recommendations, depending on their intervention group (e.g., 20-min walk every day; eat legumes 3 times per week) and develop a concrete plan to attain each goal (e.g., walk with a friend in the mall; learn to make lentil soup and hummus). The facilitator will guide the participants to consider potential barriers to goal achievement (e.g., cost of food, poor weather, fatigue), and problem solve ways to overcome them. In consequent sessions, participants will be guided to monitor their progress (e.g., did the plan work? what should be changed?) and iterate their plan as needed. The last month of goal training will focus on developing goals and plans for the follow-up period so that behavior change gains can be integrated into daily life. Those who are not in DIET or EX interventions will not receive Goal-Setting training, but will receive additional ED instead.

### Promoting adherence and retention

To promote retention and reduce the incidence of dropouts over the 12-month follow-up period, the intervention coordinator will remain in close contact with the participants and be available by phone to discuss potential issues. The team will also contact participants in advance of their 12-month follow-up assessments. Participants who withdraw from the study will be invited to attend their follow-up assessments.

### Primary outcome measures

#### Feasibility: recruitment, adherence, retention

i) Recruitment.

A research coordinator will be responsible for logging recruitment activities as participants contact the study email or phone number. The research coordinator will document the following information of each participant: name, phone number, email, date of first contact, how they heard of the study, and whether they passed telephone screening. For participants that pass initial screening, they will be scheduled to meet with an assessor for a second screening and to provide consent. Upon completing the second screening session, the research coordinator will document whether they passed or failed screening. Recruitment feasibility will be determined by the average number of participants recruited per month (target: ≥ 9 participants per month).

ii) Adherence.

Instructors will keep a detailed attendance record for each component of each session (EX/STRETCH and DIET/ED). The target is ≥ 75% of EX and ≥ 75% of DIET sessions completed [66]. Adherence will be measured through subjective participation scores that are completed by the instructor and volunteer of each session, as well as by self-reported RPE ratings provided each session by participants in the EX groups. For each EX/STRETCH and DIET/ED component, participants will be confidentially scored on a scale from 1 to 3. A score of 3 indicates that the participant actively followed along with all exercises/stretches or DIET/ED content and participated in exercises/stretches or listened or contributed greater than 80% of the session. A score of 2 indicates that a participant engaged/contributed to 50–80% of the session and a 1 indicates that a participant engaged in less than 50% of the session. Self-reported attendance records for the independent sessions will also be collected, noting RPE rating (for EX groups), and any symptoms experienced during EX and STRETCH. For diet quality specifically, adherence will be measured through adherence to the Brain Health Food Guide recommendations and will be assessed using the Eating Pattern Self-Assessment (EPSA) [54].

iii) Retention.

Patients will be considered “non-completers” upon confirmation from a participant that they no longer wish to participate or are unable to participate. When a participant drops out of the study, the instructor will inform the research coordinator who will document withdrawals. The research coordinator will categorize reasons for non-completion as medical or non-medical. The feasibility target for retention is defined as ≥ 80% of participants with complete 6-month assessments.

### Secondary outcome measures

Each participant will complete two assessments at baseline (t_1_), post-intervention (t_2_), and 6 months post-intervention (t_3_) (see Fig. 1 for overview). All assessments will take place over Zoom® with a research assistant or coordinator who is blinded to group allocation.

At virtual assessment 1 (55 min), the following will be assessed: (1) Demographics: age, sex, gender, race and ethnic origins, education, marital status, type of residence, geography of residence (urban/rural), socioeconomic status; (2) Medical and mental health history and current use of medications; (3) Technology proficiency assessed using 10 questions adapted from AGE-WELL’s Canadian Attitudes toward Technology and Aging Questionnaire [67]; (4) Functional strength assessed with the 5-time sit-to-stand test, which is a validated test of lower body function and strength among older adults [68]. The participant will be timed as they stand from a seated position 5 times using a chair of standard height (same chair at each assessment); (5) Physical activity assessed using the Physical Activity Scale for the Elderly (PASE) which is valid among older adults aged 65–100 years and will be used to characterize habitual physical activity, and maintenance of physical activity levels during the post-intervention period [69]; (6) Health-related quality of life (QOL) measured using the SF-36 questionnaire [70], which will be used to develop weightings for quality-adjusted life-years (for the full-scale trial). The SF-36 also includes 8-scale scores that can be grouped into physical health QOL and mental health QOL. The SF-36 has been shown to be a valid and practical instrument to assess the quality of life of older adults living at home [71]; (7) Psychological health including depressive symptoms measured using the Center for Epidemiological Studies-Depression scale (CES-D) [72] and perceived anxiety measured using the Geriatric Anxiety Inventory (GAI) [73]. The CES-D has demonstrated high validity and reliability among older adults [72, 74]. The GAI has demonstrated a strong ability to identify geriatric anxiety, with studies reporting a high internal consistency and validity of the questionnaire [73, 75]; (8) Waist circumference assessed as a simple indicator of vascular risk [76]. The assessor will oversee the measurement, ensuring the tape measure is in the correct position. This measurement will be taken a second time to ensure accuracy, with both values will be recorded and an average of both values being used; (9) Diet quality measured using the Eating Pattern Self-Assessment (EPSA) which collects self-reported intake of the 15 targeted food items from the BHFG. In addition to t_1_, t_2_, and t_3_, the EPSA will also be administered monthly to participants in the DIET group to help with goal setting, while it will be administered mid-way through the program for participants in the ED groups. At post-intervention, participants will complete a survey to gather feedback about their experience in the program.

At virtual assessment 2 (30 min), executive function will be assessed by a composite measure of executive function, consisting of the Cambridge Brain Sciences (CBS) Double Trouble (Stroop), Monkey Ladder (visuospatial working memory), and Feature Match (attention speed) tasks [77]. Memory will be assessed by delayed recall on the Rey Auditory Verbal Learning Test (RAVLT) [78] and the CBS Paired Associates task [77]. These tests were chosen as they measure the cognitive domains of interest and have been validated for remote delivery [79–81].

### Sample size

A recent meta-analysis of exercise on cognition found Cohen’s *d* effect sizes between 0.27 and 0.36, dependent on combinations of exercise mode, frequency, duration, and outcome [16]. Effect sizes for diet interventions are highly variable depending on the intensity of the intervention. The most similar published diet intervention was in a factorial RCT of exercise and diet (without a goal-setting component) that found an effect size of 0.32 for exercise and 0.30 for diet [82]. The intensity of the current study intervention is higher than in this prior study, and it is expected that the goal-setting will increase adherence. However, virtual delivery may weaken adherence. As a result, an effect size of 0.30 was chosen for sample size estimates for the full-scale trial.

For the full-scale trial, it is estimated that 88 participants are needed per intervention arm (total of 364 participants) to detect EX (*d* = 0.3) and DIET (*d* = 0.3) effects with an alpha level of 5% and 80% power. With 10% dropout, 404 participants are needed for a full-scale trial over 3.5 years. As a result, for this feasibility trial, 140 participants will be recruited over 1.25 years to test the feasibility of the rate of recruitment that would be required for the full trial. Interim analysis of feasibility will be performed after the first three waves complete the 6-month assessment.

This sample size should also be appropriate for measuring feasibility outcomes. To confirm the sample size was sufficient, we considered a study adherence as a dichotomous outcome with a binomial distribution, where each participant will either complete the 6-month assessment (1) or not (0). With a sample size of 140, anticipated adherence of 80% can be estimated to within a 95% confidence interval from 72% to 86%.

### Data handling

Investigators and study staff will collect only the information needed for this study. Data will be stored in password protected files on computers, and in locked rooms. Data related to questionnaires will be collected using Qualtrics software (August 2023) and stored on an encrypted drive. All personal identifying information will be removed from the data and will be replaced with an ID code. Following completion of the study, data will be made available on an open science platform (platform yet to be determined, with participant consent) so that other researchers will have the opportunity to validate and replicate the results. All data shared in an open science framework will be de-identified and only study staff will be able to link this data to any personal identifying information.

For the memory and learning tests, there may be audio recordings to ensure that the researcher scores the test results accurately. These tests will also be saved with an ID code so that names cannot be identified and they will be stored until data analysis is complete.

### Safety and monitoring procedures of adverse events

To minimize the risk of adverse events, individuals must be screened safe to participate in moderate exercise using the CSEP Get Active Questionnaire [50] or have physician approval to engage in moderate intensity exercise without in-person supervision. The risk of adverse events is minimized by having exercise prescription completed by a CEP, by monitoring symptoms at each session, and through appropriate emergency protocols. Procedures are in place to monitor adverse events that participants may experience during the intervention. Grading and categorization of adverse events will be conducted using the Common Terminology Criteria for Adverse Events [83]. Participants will be queried about adverse events at each session and study visit. In situations where a participant reports exercise-related changes, such as muscle soreness and joint pain, the CEP will meet with the participant individually to gain further information about the changes. The CEP will determine whether the participant is safe to continue to exercise (although with modifications) or whether they should seek further medical attention. If a participant is deemed not safe to continue exercising, the CEP will follow-up with the participant weekly to determine when it is safe for them to re-join (if necessary, study physicians will be consulted in this process). For all adverse events that occur during the study, research staff are instructed to contact the University of Waterloo and Baycrest Research Ethics Boards. Adverse events for this study are defined as any undesirable experience that occurs to a participant during the 6-month intervention, regardless of whether they are perceived to be related to the intervention, and whether they are an expected consequence of the intervention. For adverse events that occur which are perceived to be related to the intervention, study physicians will be alerted. If it is unclear whether an adverse event is study related, the event and the conditions will be shared with study physicians.

### Analysis

Analysis of primary objectives related to feasibility outcomes will be descriptive. “Success” will be based on predetermined criteria outlined in “Objectives and hypothesis” which included: (1) Recruitment: ≥ 9 participants/month over 1.25 years; (2) Adherence: (a) ≥ 75% of Aerobic and Resistance Exercise (EX) sessions and ≥ 75% of Diet Counseling (DIET) sessions are completed; (b) improvement in diet quality at 6 months compared to baseline; (3) Retention: ≥ 80% of randomized participants complete the 6-month assessment of executive function. Overall, “success” will indicate that the protocol is adequately robust to proceed to a large RCT with small or no adaptations, and failure to meet these targets indicating a need for substantial change before proceeding. Preliminary analyses of feasibility outcomes will be conducted at the end of year 2 by an independent statistician when the first three waves complete their 6-month assessment. If the data support feasibility, the research team will apply for funding to roll directly into the definitive trial.

Full analyses will be conducted once all participants have completed their 12-month follow-up assessments. Analyses will explore whether feasibility outcomes vary by gender as research suggests that exercise and diet preferences vary by gender [45, 84]. Feasibility outcomes will be described among women and men, and among non-binary and other gender groups if possible. Analyses will also explore whether other demographics, geographical location, or technology access predict adherence. Characteristics of the participants who withdraw and reasons for withdrawal will be analyzed and intent-to-treat analyses will be used. Individuals who withdraw from the study will be invited to take part in post-intervention and 6-month post-intervention assessments and a sensitivity analysis will be completed. In addition, effect sizes for EX and DIET will be determined for the future, full-scale trial by gender. An analysis of effect sizes will be conducted from both intention-to-treat and per protocol analyses (to understand potential effectiveness in relation to adherence).

## Discussion

This protocol summarizes a feasibility study of a 6-month virtual, factorial trial of exercise and nutrition among individuals with SCD. This study will inform the suitability of a full-scale trial. Results will also be useful to inform exercise and lifestyle interventions in research and practice, giving insights into feasibility and reach by geography and demographics. Although the frequency of virtual exercise and lifestyle trials increased during the COVID-19 pandemic, few trials recruited broadly from the community as opposed to from established research databases and participant pools. This study will give insights into the feasibility of more generalizable recruitment tactics.

The entirely virtual design will allow recruitment from a large geographic area, including individuals who rarely have access to clinical trials due to geographic region or transportation. The uptake of technology and virtual software (e.g., Zoom®) has accelerated during and after the COVID-19 pandemic [35, 85, 86], enabling reach of interventions to people with few local resources. The feasibility results of this study will inform both a future trial and broader research and practice initiatives.

The trial interventions are grounded in best evidence and practice. The interventions were informed by researchers and practitioners (exercise physiologists, kinesiologists, dietitians, occupational therapists) with subject-matter and practice expertise in exercise, diet, and goal-training. The EX intervention aligns with protocols and guidelines for physical activity for brain health [16, 87]. The DIET intervention is grounded in the “Brain Health Food Guide” recommendations, which is based on single nutrients and overall dietary patterns associated with brain health [26, 55–58]. Furthermore, the DIET intervention exceeds the intensity of prior diet interventions for brain health by combining dietary education with goal setting that has shown to be feasible and effective in other clinical trials, including a pilot study by the research team [51]. Lastly, the inclusion of active control groups that closely equate intervention intensity in terms of time and social interactions, controlling for intervention elements outside of exercise, healthy eating, and goal-training, is another strength. Overall, the use of active controls will result in a conservative estimate of the intervention effect.

However, there are challenges associated with this trial. All assessments will be completed virtually, which is not the gold standard for cognitive tests or fitness assessments. Subjective participation scores measuring adherence may also be biased. Inevitably, technology difficulties will arise, which could impact participants’ adherence and satisfaction, as well as data collection. Furthermore, it is possible that individuals who are uncomfortable with technology and those with lower levels of technology proficiency may be less inclined to join the study despite tablets and/or internet access being provided. As a result, there is a potential for selection bias and the findings not being generalizable to the older adult population.

In conclusion, the results of this study will inform whether it is feasible to move forward with a definitive trial, while also informing future virtual programs and services. If the interventions are feasible and beneficial, this study model could scale up and spread quickly to reach at-risk individuals for the purpose of dementia risk reduction.

### Trial status

Participant recruitment began in July 2023. The first session of the intervention began in October 2023 and the last session of the intervention is scheduled to finish in June 2025.

## Supplementary Information

Additional file 1. Supplementary materials

Additional file 2. SPIRIT checklist

## Data Availability

The dataset(s) supporting the conclusions of this article will be made available on an open science platform (specific platform yet to be determined).

## References

[1] World Health Organization. Dementia. 2023. Available from: https://www.who.int/news-room/fact-sheets/detail/dementia. Cited 2023 Nov 1.

[2] World Health Organization. Global action plan on the public health response to dementia 2017–2025. Available from: https://www.who.int/publications/i/item/global-action-plan-on-the-public-health-response-to-dementia-2017---2025. Cited 2023 Nov 1.

[3] Livingston G, Sommerlad A, Orgeta V, Costafreda SG, Huntley J, Ames D, Ballard C, Banerjee S, Burns A, Cohen-Mansfield J, Cooper C, Fox N, Gitlin LN, Howard R, Kales HC, Larson EB, Ritchie K, Rockwood K, Sampson EL, Samus Q, Schneider LS, Selbæk G, Teri L, Mukadam N. Dementia prevention, intervention, and care. Lancet. 2017;390(10113):2673–734. 10.1016/S0140-6736(17)31363-6. PMID: 28735855. 28735855 10.1016/S0140-6736(17)31363-6

[4] Centers for Disease Control and Prevention. Subjective cognitive decline — a public health issue. 2019. Available from: https://www.cdc.gov/aging/data/subjective-cognitive-decline-brief.html. Cited 2023 Nov 14.

[5] Statistics Canada. Seniors and aging statistics. 2020. Available from: https://www.statcan.gc.ca/eng/subjects-start/seniors_and_aging. Cited 2023 Nov 1.

[6] Cheng YW, Chen TF, Chiu MJ. From mild cognitive impairment to subjective cognitive decline: conceptual and methodological evolution. Neuropsychiatr Dis Treat. 2017;13:491. 10.2147/NDT.S123428. PMID: 28243102. 28243102 10.2147/NDT.S123428 PMC5317337

[7] Jessen F, Amariglio RE, Van Boxtel M, Breteler M, Ceccaldi M, Chételat G, et al. A conceptual framework for research on subjective cognitive decline in preclinical Alzheimer’s disease. Alzheimers Dement. 2014;10(6):844–52. 10.1016/j.jalz.2014.01.001. PMID: 2479888. 24798886 10.1016/j.jalz.2014.01.001 PMC4317324

[8] Gomez-Ramirez J, Avila-Villanueva M, Fernandez-Blazquez MA. Selecting the most important self-assessed features for predicting conversion to mild cognitive impairment with random forest and permutation-based methods. Nat Scientific Reports. 2020;10:20630. 10.1038/s41598-020-77296-4. 10.1038/s41598-020-77296-4 PMC7692490 33244011

[9] Parfenov VA, Zkharov VV, Kabaeva AR, Vakhnina NV. Subjective cognitive decline as a predictor of future cognitive decline: a systematic review. Dement Neuropsychol. 2020;14(3):248–57. 32973979 10.1590/1980-57642020dn14-030007 PMC7500809

[10] Tang-Wai DF, Smith EE, Bruneau MA, Burhan AM, Chatterjee A, Chertkow H, et al. CCCDTD5 recommendations on early and timely assessment of neurocognitive disorders using cognitive, behavioral, and functional scales. Alzheimers Dement (N Y). 2020;6(1):e12057. 10.1002/trc2.12057. PMID: 33209972. 33209972 10.1002/trc2.12057 PMC7657153

[11] Rotenberg S, Leung C, Quach H, Anderson ND, Dawson DR. Occupational performance issues in older adults with subjective cognitive decline. Disabil Rehabil. 2021:1–8. 10.1080/09638288.2021.1916626. Epub ahead of print. PMID: 33989108. 10.1080/09638288.2021.1916626 33989108

[12] Samadi M, Moradi S, Moradinazar M, Mostafai R, Pasdar Y. Dietary pattern in relation to the risk of Alzheimer’s disease: a systematic review. Neurol Sci. 2019;40(10):2031–43. 10.1007/s10072-019-03976-3. PMID: 31240575. 31240575 10.1007/s10072-019-03976-3

[13] Aridi YS, Walker JL, Wright ORL. The association between the Mediterranean dietary pattern and cognitive health: a systematic review. Nutrients. 2017;9(7):674. 10.3390/nu9070674. Published 2017 Jun 28. PMID: 28657600. 28657600 10.3390/nu9070674 PMC5537789

[14] Beckett MW, Ardern CI, Rotondi MA. A meta-analysis of prospective studies on the role of physical activity and the prevention of Alzheimer’s disease in older adults. BMC Geriatr. 2015;15:9. 10.1186/s12877-015-0007-2. PMID: 25887627. 25887627 10.1186/s12877-015-0007-2 PMC4333880

[15] Blondell SJ, Hammersley-Mather R, Veerman JL. Does physical activity prevent cognitive decline and dementia?: a systematic review and meta-analysis of longitudinal studies. BMC Public Health. 2014;14: 510. 10.1186/1471-2458-14-510. PMID: 24885250. 24885250 10.1186/1471-2458-14-510 PMC4064273

[16] Northey JM, Cherbuin N, Pumpa KL, Smee DJ, Rattray B. Exercise interventions for cognitive function in adults older than 50: a systematic review with meta-analysis. Br J Sports Med. 2018;52(3):154–60. 10.1136/bjsports-2016-096587. PMID: 28438770. 28438770 10.1136/bjsports-2016-096587

[17] Radd-Vagenas S, Duffy SL, Naismith SL, Brew BJ, Flood VM, Fiatarone Singh MA. Effect of the Mediterranean diet on cognition and brain morphology and function: a systematic review of randomized controlled trials. Am J Clin Nutr. 2018;107(3):389–404. 10.1093/ajcn/nqx070. PMID: 29566197. 29566197 10.1093/ajcn/nqx070

[18] Loughrey DG, Lavecchia S, Brennan S, Lawlor BA, Kelley ME. The impact of the Mediterranean diet on the cognitive functioning of healthy older adults: a systematic review and meta-analysis. Adv Nutr. 2017;8(4):571–86. 10.3945/an.117.015495. PMID: 28710144. 28710144 10.3945/an.117.015495 PMC5502874

[19] Erickson K, Hillman C, Stillman CM, Ballard RM, Bloodgood B, Conroy DE, et al. For 2018 physical activity guidelines advisory committee. Physical activity, cognition, and brain outcomes: a review of the 2018 physical activity guidelines. Med Sci Sports Exerc. 2019;51(6):1242–51. 10.1249/MSS.0000000000001936. PMID: 3109508. 31095081 10.1249/MSS.0000000000001936 PMC6527141

[20] Erickson KI, Voss MW, Prakash RS, Basak C, Szabo A, Chaddock L, et al. Exercise training increases size of hippocampus and improves memory. Proc Natl Acad Sci USA. 2011;108(7):3017–22. 10.1073/pnas.1015950108. PMID: 21282661. 21282661 10.1073/pnas.1015950108 PMC3041121

[21] Voss MW, Prakash RS, Erickson KI, Basak C, Chaddock L, Kim JS, et al. Plasticity of brain networks in a randomized intervention trial of exercise training in older adults. Front Aging Neurosci. 2010;2: 32. 10.3389/fnagi.2010.00032. PMID: 20890449. 20890449 10.3389/fnagi.2010.00032 PMC2947936

[22] Colcombe SJ, Erickson KI, Scalf PE, Kim JS, Prakash R, McAuley E, et al. Aerobic exercise training increases brain volume in aging humans. J Gerontol A Biol Sci Med Sci. 2006;61(11):1166–70. 10.1093/gerona/61.11.1166. PMID: 17167157. 17167157 10.1093/gerona/61.11.1166

[23] van den Brink AC, Brouwer-Brolsma EM, Berendsen AAM, van de Rest O. The Mediterranean, Dietary Approaches to Stop Hypertension (DASH), and Mediterranean-DASH Intervention for Neurodegenerative Delay (MIND) diets are associated with less cognitive decline and a lower risk of Alzheimer’s disease-a review. Adv Nutr. 2019;10:1040–65. 10.1093/advances/nmz054. 31209456 10.1093/advances/nmz054 PMC6855954

[24] Zamroziewicz MK, Barbey AK. Role of the Mediterranean diet in the brain and neurodegenerative diseases. London: Academic Press; 2018. The Mediterranean diet and healthy brain aging: innovations from nutritional cognitive neuroscience; pp. 17–33.

[25] Fu J, Tan LJ, Lee JE, Shin S. Association between the Mediterranean diet and cognitive health among healthy adults: a systematic review and meta-analysis. Front Nutr. 2022;9: 946361. 10.3389/fnut.2022.946361. 35967772 10.3389/fnut.2022.946361 PMC9372716

[26] Martínez-Lapiscina EH, Clavero P, Toledo E, Estruch R, Salas-Salvadó J, San Julián B, et al. Mediterranean diet improves cognition: the PREDIMED-NAVARRA randomised trial. J Neurol Neurosurg Psychiatry. 2013;84(12):1318–25. 10.1136/jnnp-2012-304792. PMID: 23670794. 23670794 10.1136/jnnp-2012-304792

[27] Blumenthal JA, Smith PJ, Mabe S, Hinderliter A, Lin PH, Liao L, et al. Lifestyle and neurocognition in older adults with cognitive impairments: a randomized trial. Neurology. 2019;92(3):e212–23. 10.1212/WNL.0000000000006784. PMID: 30568005. 30568005 10.1212/WNL.0000000000006784 PMC6340382

[28] Knight A, Bryan J, Wilson C, Hodgson JM, Davis CR, Murphy KJ. The Mediterranean diet and cognitive function among healthy older adults in a 6-month randomised controlled trial: the MedLey Study. Nutrients. 2016;8(9): 579. 10.3390/nu8090579. PMID: 27657119. 27657119 10.3390/nu8090579 PMC5037563

[29] Valls-Pedret C, Sala-Vila A, Serra-Mir M, Corella D, De la Torre R, Martínez-González MÁ, et al. Mediterranean diet and age-related cognitive decline: a randomized clinical trial. JAMA Int Med. 2015;175(7):1094–103. 10.1001/jamainternmed.2015.1668. PMID: 25961184. 10.1001/jamainternmed.2015.1668 25961184

[30] Marseglia A, Xu W, Fratiglioni L, Fabbri C, Berendsen AA, Bialecka-Debek A, et al. Effect of the NU-AGE diet on cognitive functioning in older adults: a randomized controlled trial. Front Physiol. 2018;9: 349. 10.3389/fphys.2018.00349. PMID: 29670545. 29670545 10.3389/fphys.2018.00349 PMC5893841

[31] Otaegui-Arrazola A, Amiano P, Elbusto A, Urdaneta E, Martínez-Lage P. Diet, cognition, and Alzheimer’s disease: food for thought. Eur J Nutr. 2014;53(1):1–23. 10.1007/s00394-013-0561-3. PMID: 28438770. 23892520 10.1007/s00394-013-0561-3

[32] Goran MI, Poehlman ET. Endurance training does not enhance total energy expenditure in healthy elderly persons. Am J Physiol. 1992;263:E950–7. 10.1152/ajpendo.1992.263.5.E950. PMID: 144312. 1443128 10.1152/ajpendo.1992.263.5.E950

[33] Government of Canada. Canadian Radio-Television and Telecommunications Commission. Communications monitoring report – LTE and broadband availability. 2020. Available from: https://crtc.gc.ca/eng/publications/reports/policyMonitoring/2020/cmr4.htm.

[34] Government of Canada. Canadian Radio-Television and Telecommunications Commission. Broadband fund: closing the digital divide in Canada. 2021. Available from: https://crtc.gc.ca/eng/internet/internet.htm.

[35] Age-Well. COVID-19 has significantly increased the use of many technologies among older Canadians: poll. 2020. Available from: https://agewell-nce.ca/archives/10884. Cited 2023 Nov 8.

[36] Neudorf B, Dinh C, Barnes V, Stergiou-Dayment C, Middleton L. Enhancing Minds in Motion® as a virtual program delivery model for people living with dementia and their care partners. PLoS One. 2024;19(1):e0291166. 10.1371/journal.pone.0291166. PMID: 38241269. 38241269 10.1371/journal.pone.0291166 PMC10798436

[37] Maresova P, Krejcar O, Maskuriy R, Bakar NAA, Selamat A, Truhlarova Z, Horak J, Joukl M, Vítkova L. Challenges and opportunity in mobility among older adults - key determinant identification. BMC Geriatr. 2023;23(1):447. 10.1186/s12877-023-04106-7. PMID: 37474928. 37474928 10.1186/s12877-023-04106-7 PMC10360303

[38] Hobson N, Dupuis SL, Giangregorio LM, Middleton LE. Perceived facilitators and barriers to exercise among older adults with mild cognitive impairment and early dementia. J Aging Phys Act. 2020;28(2):208–18. 10.1123/japa.2019-0010. PMID: 31621645. 31621645 10.1123/japa.2019-0010

[39] Pike K, Moller CI, Bryant C, Farrow M, Dao DP, Ellis KA. Examination of the feasibility, acceptability, and efficacy of the online personalised training in memory strategies for everyday program for older adults: single-arm pre-post trial. J Med Internet Res. 2023A;25:e41712. 10.2196/41712. 37079356 10.2196/41712 PMC10160943

[40] da Silva WA, Martins VF, Haas AN, Gonçalves AK. Online exercise training program for Brazilian older adults: effects on physical fitness and health-related variables of a feasibility study in times of COVID-19. Int J Environ Res Public Health. 2022;19(21):14042. 10.3390/ijerph192114042. 36360923 10.3390/ijerph192114042 PMC9658741

[41] Shim M, Kavanaugh M, Lacson C, Goldstein-Levitas N, Chang H, Zhang F, Palekar N, Gonzalez A, Fisher K. Connected through movement: a feasibility study of online mindfulness-based dance/movement therapy for older adults with age-related cognitive decline during COVID-19. Aging Ment Health. 2024;28(12):1676–85. 10.1080/13607863.2024.2364754. 38910361 10.1080/13607863.2024.2364754

[42] Karkon S, O’Shea F, Doran M, McCormack H, Connolly D. Testing the feasibility and acceptability of an online ‘Fatigue and Activity Management Education for Work (FAME-W) programme’ for individuals with inflammatory arthritis. Musculoskeletal Care. 2023;21(3):815–26. 10.1002/msc.1756. 36929565 10.1002/msc.1756

[43] Li F, Harmer P, Voit J, Chou LS. Implementing an online virtual falls prevention intervention during a public health pandemic for older adults with mild cognitive impairment: a feasibility trial. Clin Interv Aging. 2021;16:973–83. 10.2147/CIA.S306431. 34079243 10.2147/CIA.S306431 PMC8164667

[44] Li F, Harmer P, Fitzgerald K, Winters-Stone K. A cognitively enhanced online Tai Ji Quan training intervention for community-dwelling older adults with mild cognitive impairment: a feasibility trial. BMC Geriatr. 2022;22(1):76. 10.1186/s12877-021-02747-0. PMID: 35078407. 35078407 10.1186/s12877-021-02747-0 PMC8787180

[45] Masella R, Malorni W. Gender-related differences in dietary habits. Clin Manage Iss. 2017;11(2):59–62. 10.7175/cmi.v11i2.1313.

[46] Westenhoefer J. Age and gender dependent profile of food choice. Forum Nutr. 2005;57:44–51. 10.1159/000083753. PMID: 15702587. 10.1159/000083753 15702587

[47] Brenkel M, Shulman K, Hazan E, Herrmann N, Owen AM. Assessing capacity in the elderly: comparing the MoCA with a novel computerized battery of executive function. Dement Geriatr Cogn Dis Extra. 2017;7(2):249–56. 10.1159/000478008. 28868068 10.1159/000478008 PMC5567119

[48] Barha CK, Davis JC, Falck RS, Nagamatsu LS, Liu-Ambrose T. Sex differences in exercise efficacy to improve cognition: a systematic review and meta-analysis of randomized controlled trials in older humans. Front Neuroendocrinol. 2017;46:71–85. 10.1016/j.yfrne.2017.04.002. PMID: 28442274. 28442274 10.1016/j.yfrne.2017.04.002

[49] Jessen F, Wolfsgruber S, Wiese B, Bickel H, Mösch E, Kaduszkiewicz H, et al. AD dementia risk in late MCI, in early MCI, and in subjective memory impairment. Alzheimers Dement. 2014;10(1):76–83. 10.1016/j.jalz.2012.09.017. PMID: 23375567. 23375567 10.1016/j.jalz.2012.09.017

[50] Canadian Society for Exercise Physiology. Get active questionnaire. 2017. Available from: https://www.csep.ca/CMFiles/GAQ_CSEPPATHReadinessForm_2pages.pdf. Cited 2023 Sept 12.

[51] Koblinsky ND, Anderson ND, F Ajwani, Parrot MD, Dawson D, Marzolini S, Oh P, Middleton L, Ferland G, Greenwood CE. Feasibility and preliminary efficacy of the Lifestyle, Exercise and Diet (LEAD) study: a cluster randomized controlled trial to determine the effects of an intensive diet intervention on brain structure in older adults at risk of dementia. 2021;8(37). 10.1186/s40814-022-00977-6. 10.1186/s40814-022-00977-6 PMC8826667 35139918

[52] Health E University. Cardiac College: resistance training exercises. 2023. Available from: https://www.healtheuniversity.ca/EN/CardiacCollege/Active/Resistance_Training/Exercises/Pages/exercises.aspx. Cited 2024 Jan 4.

[53] Borg GA. Psychophysical bases of perceived exertion. Med Sci Sport Exer. 1982;4:377–81 PMID: 7154893. 7154893

[54] CCNA. Brain health food guide. 2023. Available from: https://ccna-ccnv.ca/brain-health-food-guide-2/. Cited 2024 Jan 4.

[55] Morris MC, Tangney CC, Wang Y, et al. MIND diet slows cognitive decline with aging. Alzheimers Dement. 2015;11:1015–22 PMID: 26086182. 26086182 10.1016/j.jalz.2015.04.011 PMC4581900

[56] Sacks FM, Obarzanek E, Windhauser MM, Svetkey LP, Vollmer WM, McCullough M, et al. Rationale and design of the Dietary Approaches to Stop Hypertension trial (DASH). A multicenter controlled-feeding study of dietary patterns to lower blood pressure. Ann Epidemiol. 1995;5(2):108–18 PMID: 7795829. 7795829 10.1016/1047-2797(94)00055-x

[57] Scarmeas N, Anastasiou CA, Yannakoulia M. Nutrition and prevention of cognitive impairment. Lancet Neurol. 2018;17:1006–15. 10.1016/S1474-4422(18)30338-7. PMID: 30244829. 30244829 10.1016/S1474-4422(18)30338-7

[58] van den Brink AC, Brouwer-Brolsma EM, Berendsen AAM, van de Rest O. The Mediterranean, Dietary Approaches to Stop Hypertension (DASH), and Mediterranean-DASH Intervention for Neurodegenerative Delay (MIND) diets are associated with less cognitive decline and a lower risk of Alzheimer’s disease—a review. Adv Nutr. 2019;00:1–26. 10.1093/advances/nmz054. PMID: 31209456. 10.1093/advances/nmz054 PMC6855954 31209456

[59] Bethell J, Pringle D, Chambers LW, Cohen C, Commisso E, Cowan K, et al. Patient and public involvement in identifying dementia research priorities. J Am Geriatr Soc. 2018;66(8):1608–12. 10.1111/jgs.15453. PMID: 30084194. 30084194 10.1111/jgs.15453

[60] Dawson DR, McEwen SE, Polatajko HJ, editors. Cognitive orientation to daily occupational performance in occupational therapy: using the CO-OP approach to enable participation across the lifespan. Bethseda: AOTA Press; 2017.

[61] Dawson DR, Anderson ND, Binns MA, Bottari C, Damianakis T, Hunt A, Polatajko HJ, Zwarenstein M. Managing executive dysfunction following acquired brain injury and stroke using an ecologically valid rehabilitation approach: a study protocol for a randomized, controlled trial. Trials. 2013;14:306. 10.1186/1745-6215-14-306. 24053695 10.1186/1745-6215-14-306 PMC3849520

[62] Nelis SM, Thom JM, Jones IR, Hindle JV, Clare L. Goal-setting to promote a healthier lifestyle in later life: qualitative evaluation of the AgeWell trial. Clin Gerontol. 2018;41(4):335–45. 10.1080/07317115.2017.1416509. PMID: 29308992. 29308992 10.1080/07317115.2017.1416509 PMC5942145

[63] Michie S, Ashford S, Sniehotta FF, Dombrowski SU, Bishop A, French DP. A refined taxonomy of behaviour change techniques to help people change their physical activity and healthy eating behaviours: the CALO-RE taxonomy. Psychol Health. 2011;26(11):1479–98. 10.1080/08870446.2010.540664. PMID: 21678185. 21678185 10.1080/08870446.2010.540664

[64] Carraça E, Encantado J, Battista F, Beaulieu K, Blundell J, Busetto L, van Baak M, Dicker D, Ermolao A, Farpour-Lambert N, Pramono A, Woodward E, Bellicha A, Oppert JM. Effective behavior change techniques to promote physical activity in adults with overweight or obesity: a systematic review and meta-analysis. Obes Rev. 2021;22(Suppl 4): e13258. 10.1111/obr.13258. PMID: 33949778. 33949778 10.1111/obr.13258 PMC8365685

[65] Zaslavsky O, Su Y, Kim B, Roopsawang I, Wu KC, Renn BN. Behavior change factors and retention in dietary interventions for older adults: a scoping review. Gerontologist. 2022;62(9):e534–54. 10.1093/geront/gnab133. PMID: 34477843. 34477843 10.1093/geront/gnab133 PMC9756309

[66] Pavey T, Taylor A, Hillsdon M, Fox K, Campbell J, Foster C, Moxham T, Mutrie N, Searle J, Taylor R. Levels and predictors of exercise referral scheme uptake and adherence: a systematic review. J Epidemiol Community Health. 2012;66(8):737–44. 10.1136/jech-2011-200354. 22493474 10.1136/jech-2011-200354

[67] Age-Well. 7 in 10 Canadians over the age of 65 feel confident about technology use and 86% are online daily. 2019. Available from: https://agewell-nce.ca/archives/8713. Cited 2023 Nov 8.

[68] Mong Y, Teo TW, Ng SS. 5-Repetition sit-to-stand test in subjects with chronic stroke: reliability and validity. Arch Phys Med Rehabil. 2010;91(3):407–13. 10.1016/j.apmr.2009.10.030. 20298832 10.1016/j.apmr.2009.10.030

[69] Washburn RA, McAuley E, Katula J, Mihalko SL, Boileau RA. The physical activity scale for the elderly (PASE): evidence for validity. J Clin Epidemiol. 1999;52(7):643–51. 10.1016/s0895-4356(99)00049-9. PMID: 10391658. 10391658 10.1016/s0895-4356(99)00049-9

[70] Brazier JE, Harper R, Jones NM, O’Cathain A, Thomas KJ, Usherwood T, Westlake L. Validating the SF-36 health survey questionnaire: new outcome measure for primary care. BMJ. 1992;305(6846):160–4. 10.1136/bmj.305.6846.160. PMID: 1285753; PMCID: PMC1883187. 1285753 10.1136/bmj.305.6846.160 PMC1883187

[71] Walters SJ, Munro JF, Brazier JE. Using the SF-36 with older adults: a cross-sectional community-based survey. Age Ageing. 2001;30(4):337–43. 10.1093/ageing/30.4.337. PMID: 11509313. 11509313 10.1093/ageing/30.4.337

[72] Lewinsohn PM, Seeley JR, Roberts RE, Allen NB. Center for Epidemiological Studies-Depression Scale (CES-D) as a screening instrument for depression among community-residing older adults. Psychol and Aging. 1997;12:277–87.https://doi.org/10.1037//0882-7974.12.2.277. PMID: 9189988. 10.1037//0882-7974.12.2.277 9189988

[73] Pachana NA, Byrne GJ, Siddle H, Koloski N, Harley E, Arnold E. Development and validation of the geriatric anxiety inventory. Int Psychogeriatr. 2007;19:103–14. 10.1017/S1041610206003504. PMID: 16805925. 16805925 10.1017/S1041610206003504

[74] Beekman AT, Deeg DJ, Van Limbeek J, Braam AW, De Vries MZ, Van Tilburg W. Criterion validity of the Center for Epidemiologic Studies Depression scale (CES-D): results from a community-based sample of older subjects in the Netherlands. Psychol Med. 1997;27(1):231–5. 10.1017/s0033291796003510. PMID: 9122304. 9122304 10.1017/s0033291796003510

[75] Johnco C, Knight A, Tadic D, Wuthrich VM. Psychometric properties of the Geriatric Anxiety Inventory (GAI) and its short-form (GAI-SF) in a clinical and non-clinical sample of older adults. Int Psychogeriatr. 2015;27(7):1089–97. 10.1017/S1041610214001586. PMID: 25111285. 25111285 10.1017/S1041610214001586 PMC4501012

[76] de Koning L, Merchant AT, Pogue J, Anand SS. Waist circumference and waist-to-hip ratio as predictors of cardiovascular events: meta-regression analysis of prospective studies. Eur Heart J. 2007;28(7):850–6. 10.1093/eurheartj/ehm026. PMID: 17403720. 17403720 10.1093/eurheartj/ehm026

[77] Lowe C, Rabbitt P. Test/re-test reliability of the CANTAB and ISPOCD neuropsychological batteries: theoretical and practical issues. Cambridge neuropsychological test automated battery. International study of post-operative cognitive dysfunction. Neuropsychologia. 1998;36:915–23. 9740364 10.1016/s0028-3932(98)00036-0

[78] Schmidt M. Rey auditory verbal learning test: a handbook. Los Angeles: Western Psychological Services; 1996.

[79] Hampshire A, Highfield RR, Parkin BL, Owen AM. Fractionating human intelligence. Neuron. 2012;76(6):1225–37. 23259956 10.1016/j.neuron.2012.06.022

[80] Jagtop S, Dawson DR, Vandermorris S, Anderson ND, Davids-Brumer N, Bernick A, Rotenberg S. Known-groups and convergent validity of the telephone rey auditory verbal learning test total learning scores for distinguishing between older adults with amnestic mild cognitive impairment and subjective cognitive decline. Archives of Clinical Neuropsychology. 2021;36:626–31. 33067996 10.1093/arclin/acaa085 PMC8138831

[81] Boone KB, Sherman D, Mishler J, Daoud G, Cottingham M, Victor TL, Ziegler E, Zeller MA, Wright M. Cross-validation of RAVLT performance validity indicators and the RAVLT/RO discriminant function in a large known groups sample. Clin Neuropsychol. 2022;36(8):2342–60. 10.1080/13854046.2021.1948611. PMID: 34311662. 34311662 10.1080/13854046.2021.1948611

[82] Zheng G, Xia R, Zhou W, Tao J, Chen L. Aerobic exercise ameliorates cognitive function in older adults with mild cognitive impairment: a systematic review and meta-analysis of randomised controlled trials. Br J Sports Med. 2016;50(23):1443–50. 10.1136/bjsports-2015-095699. PMID: 27095745. 27095745 10.1136/bjsports-2015-095699

[83] US Department of Health Human Services. Common terminology criteria for adverse events (CTCAE). Version 4.0. 2009. Available from: https://evs.nci.nih.gov/ftp1/CTCAE/CTCAE_4.03/Archive/CTCAE_4.0_2009-05-29_QuickReference_8.5x11.pdf.

[84] van Uffelen JG, Khan A, Burton NW. Gender differences in physical activity motivators and context preferences: a population-based study in people in their sixties. BMC Pub Health. 2017;17(1):1–1. 10.1186/s12889-017-4540-0. PMID: 28676081. 28676081 10.1186/s12889-017-4540-0 PMC5496591

[85] Statistics Canada. Analytical studies branch research paper series: evolving internet use among Canadian seniors. 2019. Available from: https://www150.statcan.gc.ca/n1/pub/11f0019m/11f0019m2019015-eng.htm. Cited 2023 Nov 2.

[86] AARP Research. Personal tech and the pandemic: older adults are upgrading for a better online experience. 2021. Available from: https://www.aarp.org/research/topics/technology/info-2021/2021-technology-trends-older-americans.html. Cited 2023 Nov 2.

[87] Ontario Brain Institute. Physical activity and Alzheimer’s disease toolkit. 2023. Available from: https://braininstitute.ca/resources/guidelines-and-toolkits/physical-activity-and-alzheimers-disease-toolkit. Cited 2024 Feb 23.

