# Supplementary material for: Rationale and protocol of the LEAD 2.0 study: a randomized controlled trial assessing the feasibility of a virtually delivered 6-month exercise and nutrition intervention in older adults with subjective cognitive decline (SCD)
Source: Pilot Feasibility Stud. 2025 May 10;11:64. doi: 10.1186/s40814-025-01626-4 (PMC12065297; doi:10.1186/s40814-025-01626-4)
Supplement: Supplementary file 1 — Additional file 1. Supplementary materials [file 40814_2025_1626_MOESM1_ESM.docx]

Supplementary Materials

S1. Recruitment Screening Script

S2. Participant Intake Form

S3. Brain Health Food Guide

S4. Eating Pattern Self-Assessment


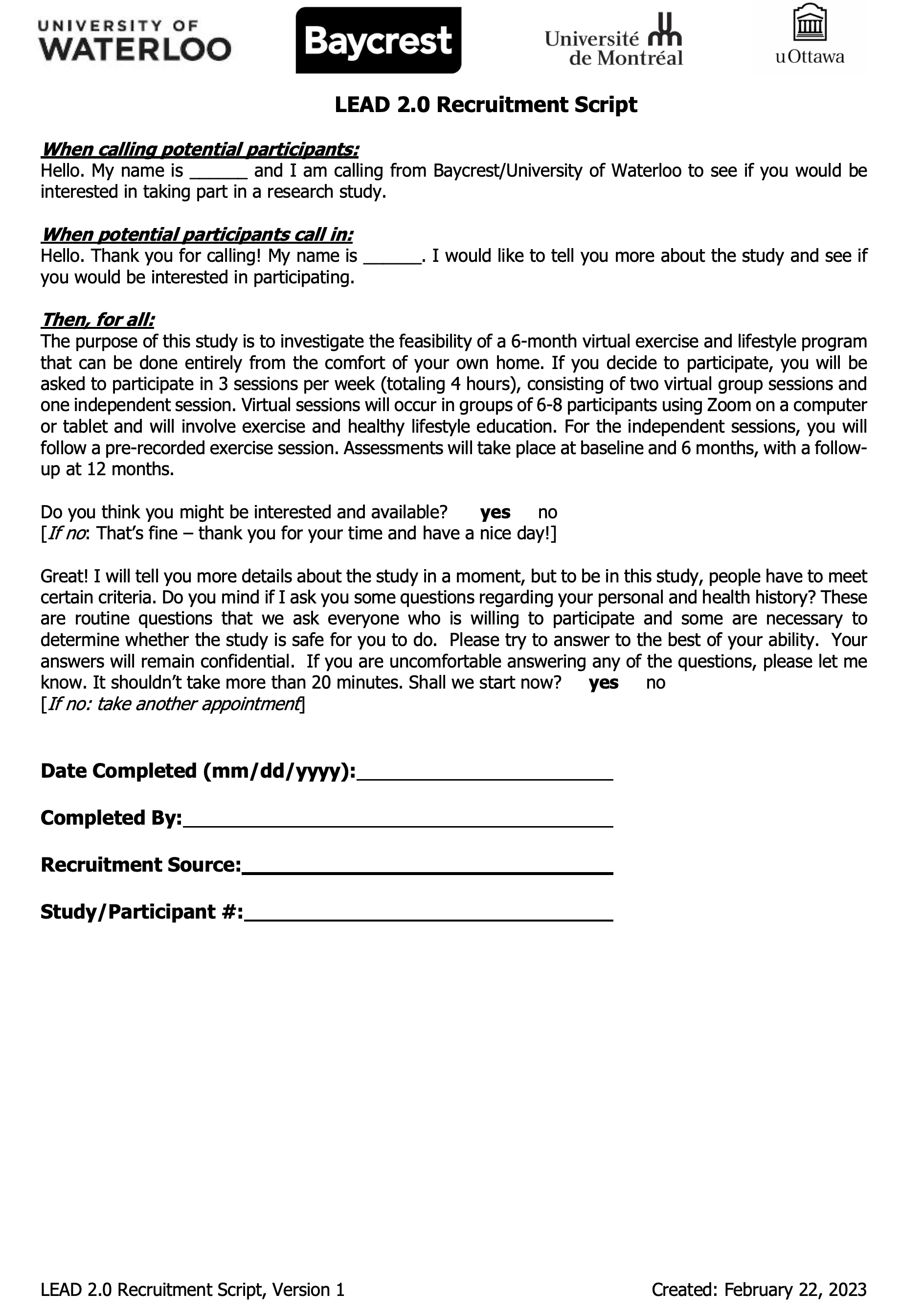
S1. Recruitment Screening Script


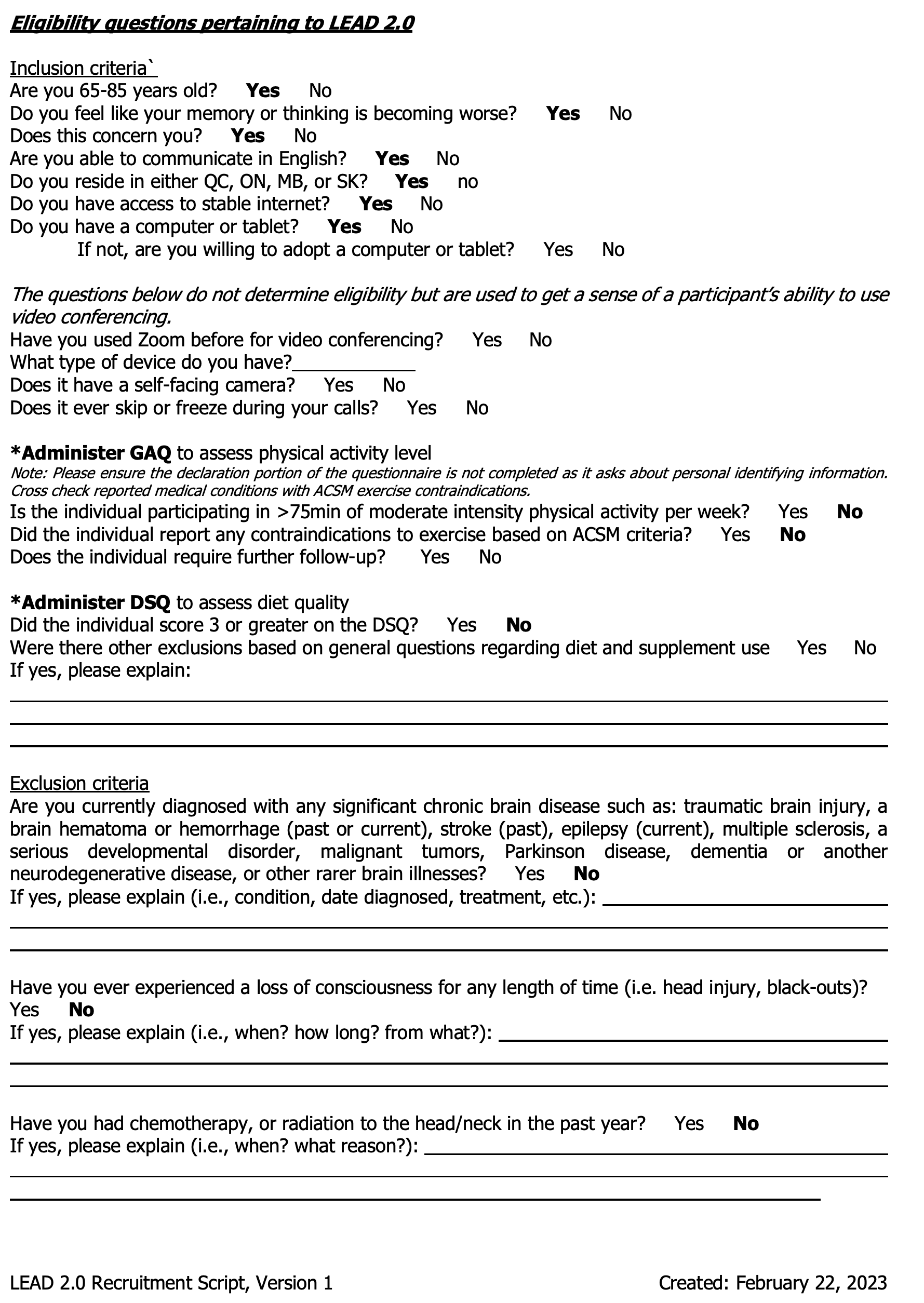


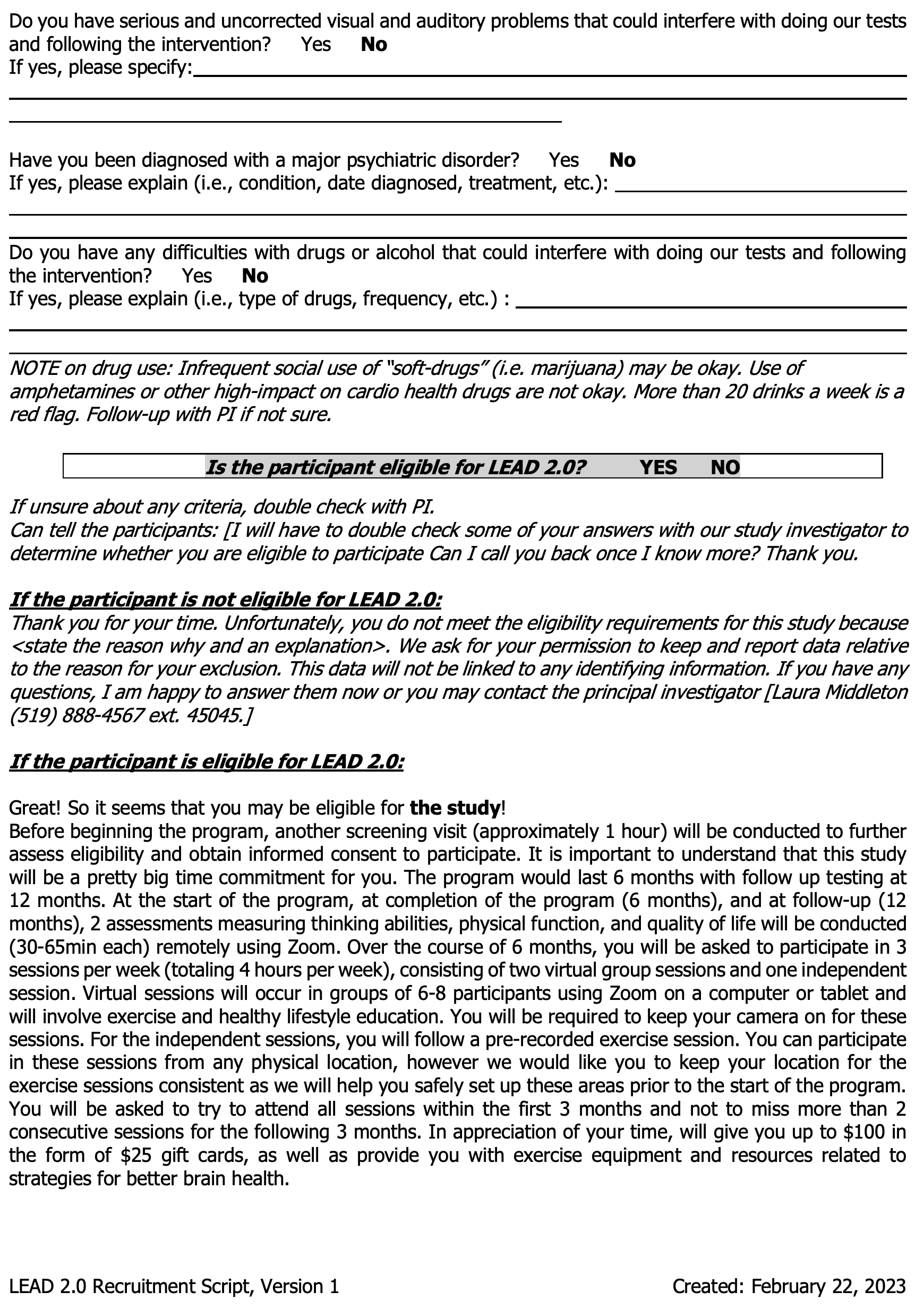


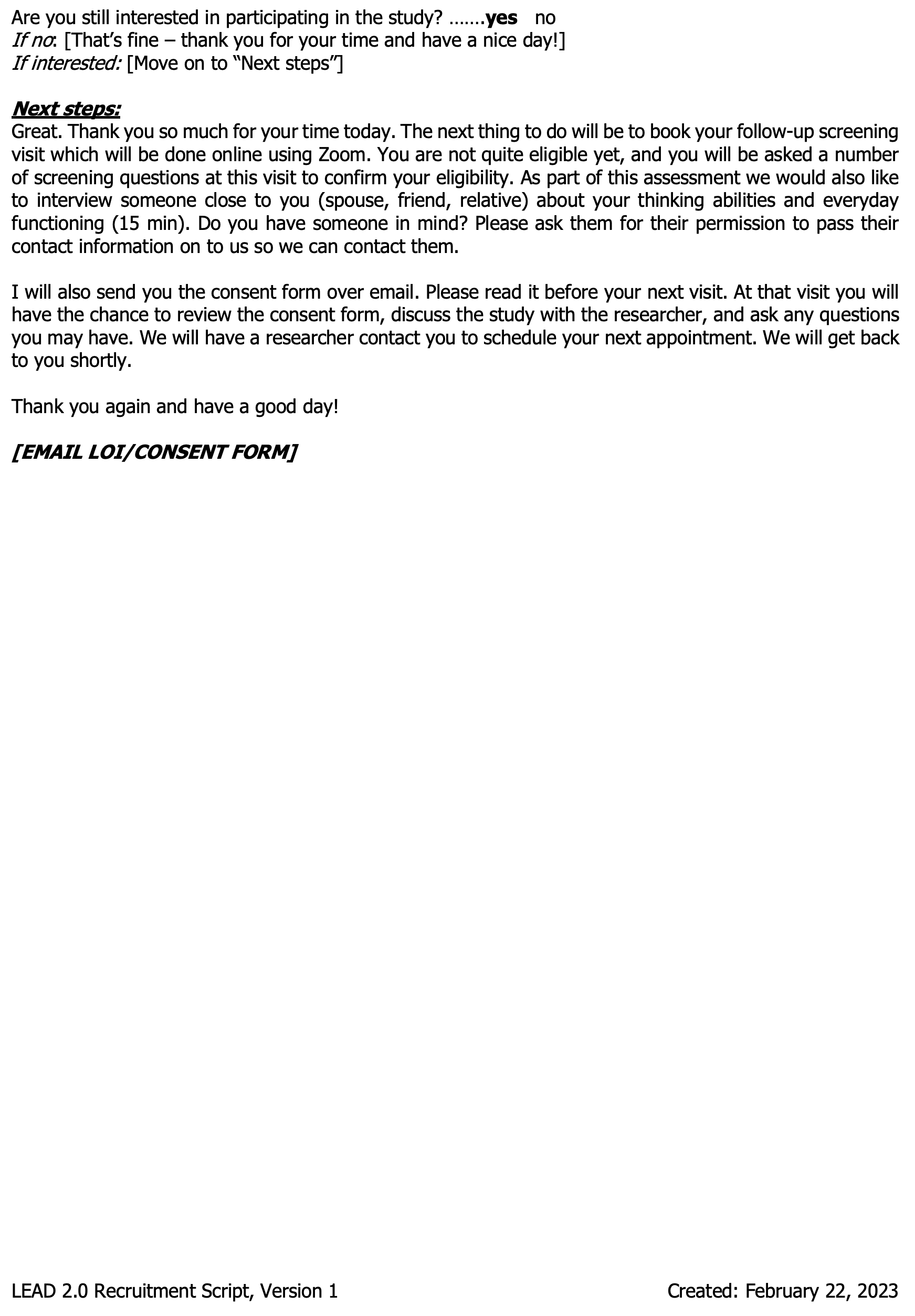


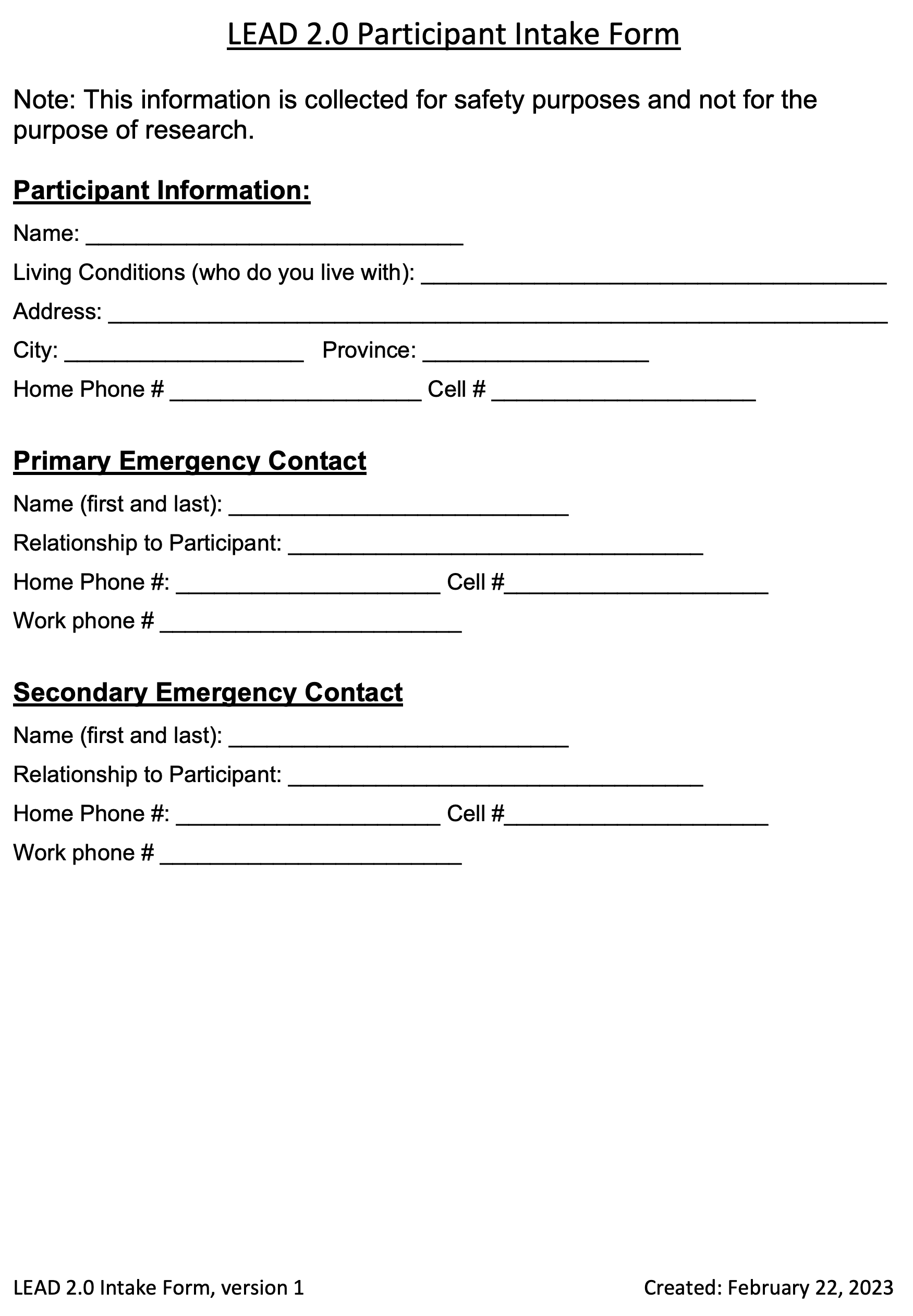
S2. Participant Intake Form


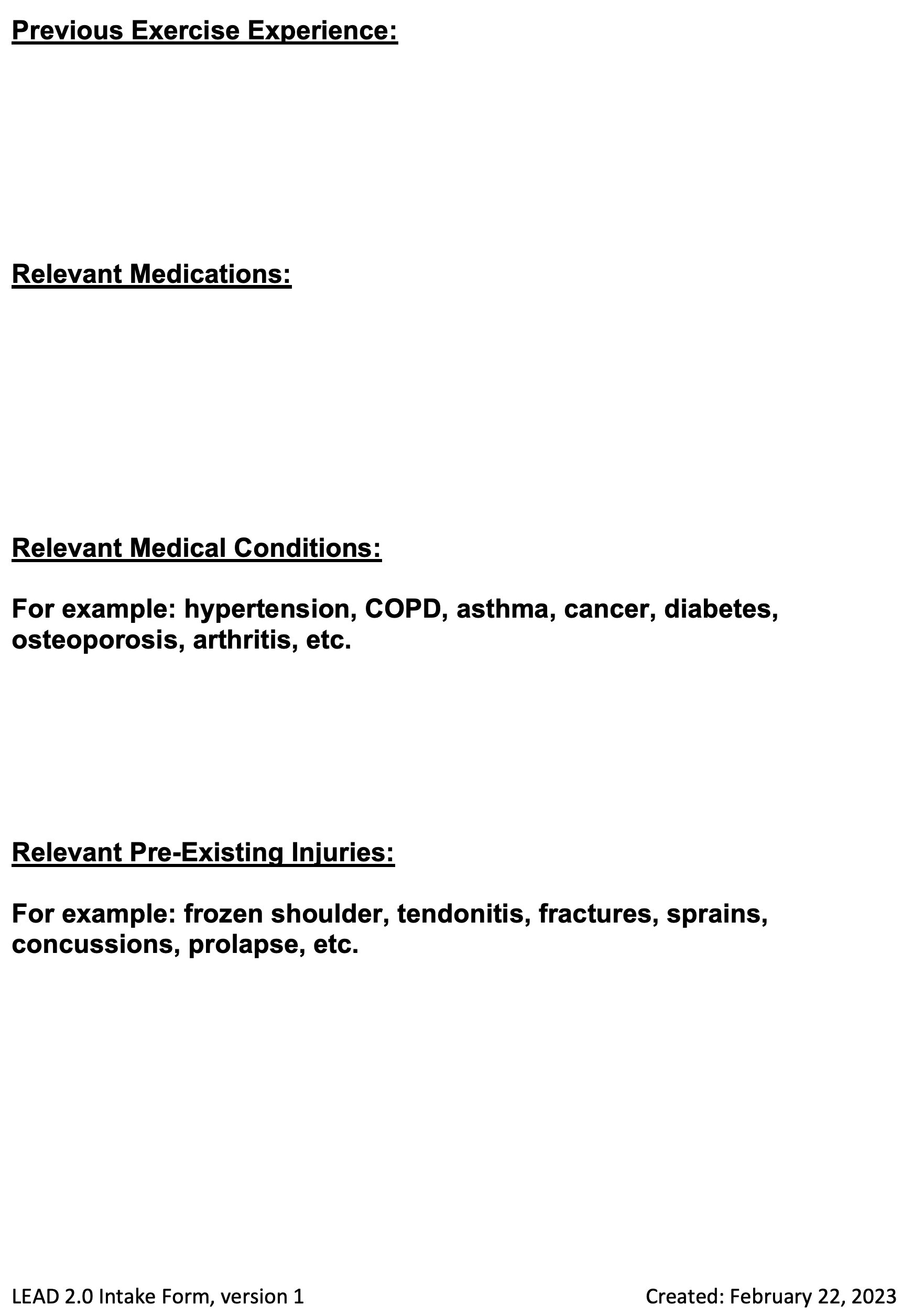


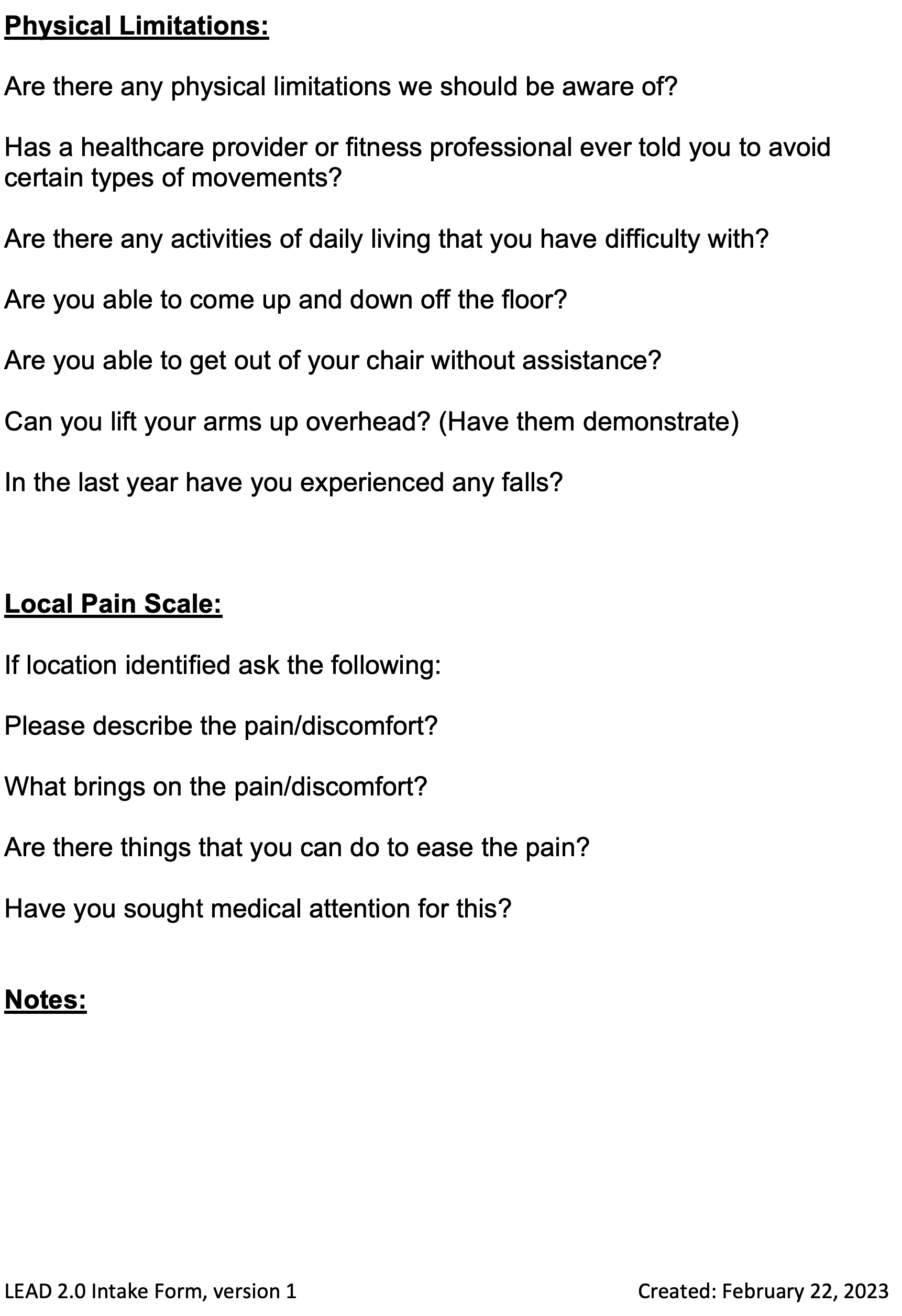


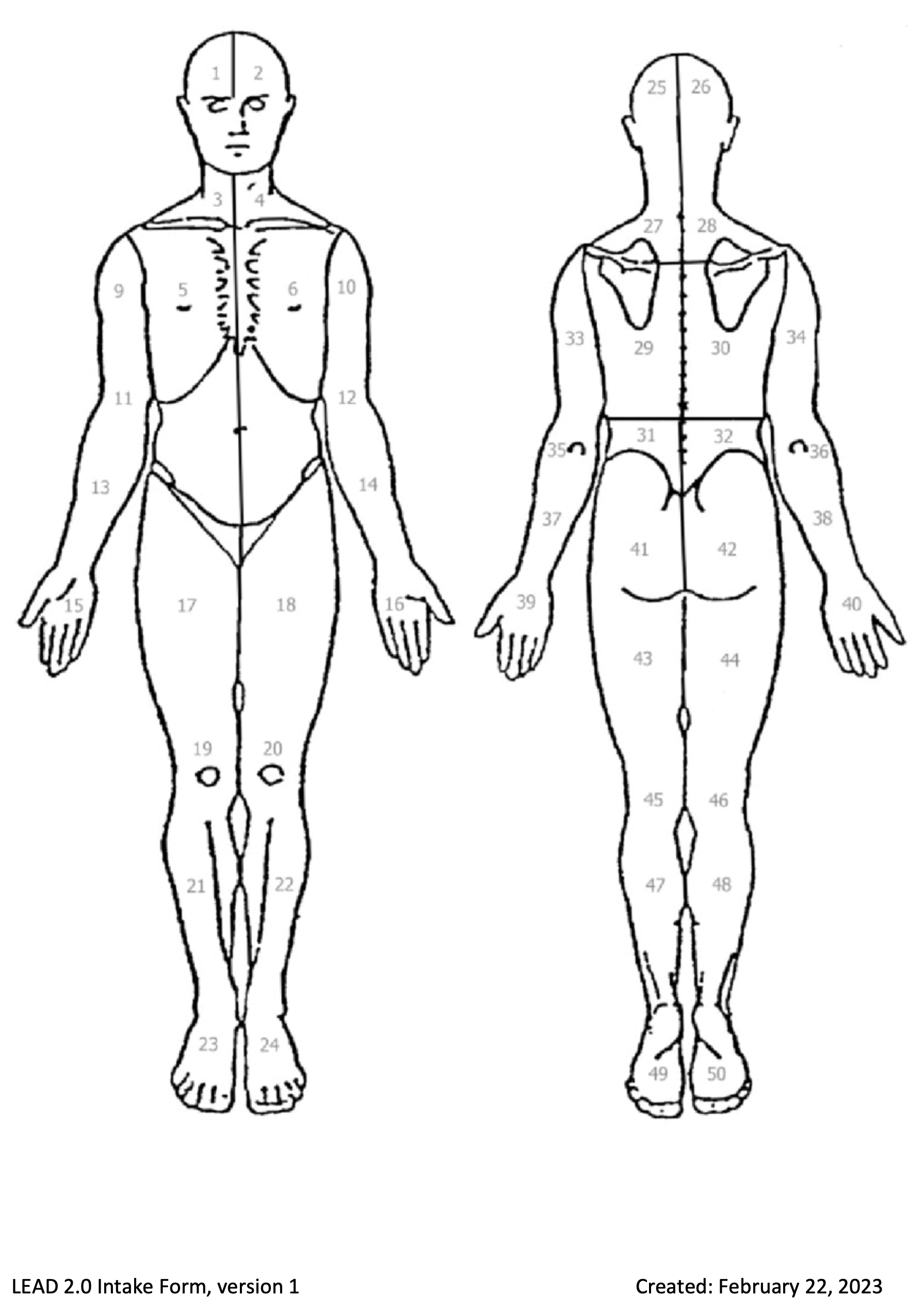

S3. Brain Health Food Guide


S4. Eating Pattern Self-Assessment
